# The parametric study of hybrid nanofluid flow with heat transition characteristics over a fluctuating spinning disk

**DOI:** 10.1371/journal.pone.0254457

**Published:** 2021-08-16

**Authors:** Xiao-Hong Zhang, Ebrahem A. Algehyne, Maryam G. Alshehri, Muhammad Bilal, Muhammad Altaf Khan, Taseer Muhammad

**Affiliations:** 1 College of Science, Hunan City University, Yiyangt, P. R. China; 2 Faculty of Science, Department of Mathematics, University of Tabuk, Tabuk, Saudi Arabia; 3 Nanotechnology Research Unit (NRU), University of Tabuk, Tabuk, Saudi Arabia; 4 Department of Mathematics, City University of Science and Information Technology, Peshawar, Pakistan; 5 Faculty of Natural and Agricultural Sciences, Institute for Groundwater Studies, University of the Free State, Bloemfontein, South Africa; 6 Department of Mathematics, College of Sciences, King Khalid University, Abha, Saudi Arabia; Stellenbosch University, SOUTH AFRICA

## Abstract

The study explored the 3D numerical solution of an unsteady *Ag-MgO*/water hybrid nanofluid flow with mass and energy transmission generated by a wavy rotating disc moving up and down. The nanofluid is generated in the context of Ag-MgO nanomaterials. Magnesium oxide and silver nanoparticles have been heavily reported to have broad-spectrum antibacterial operations among metal oxides and metals. Silver nanoparticles are without a doubt the most commonly used inorganic nanoparticles, with numerous innovations in biomaterial’s detection and antimicrobial operations. However, in current paper, the intention of the analysis is to boost thermal energy transmitting rates for a range of industrial implementations. When compared to a flat surface, energy transition is increased up to 15% due to the wavy swirling surface. The problem has been formulated as a system of PDEs, which included the Navier Stokes and Maxwell equations. Following that, the modeled equations are reduced to a dimensionless system of differential equations. The derived equations are then solved numerically using the Parametric Continuation Method (PCM). The findings are displayed graphically and debated. The geometry of a spinning disc is thought to have a positive impact on velocity and heat energy transfer. The insertion of nanostructured materials (silver and magnesium-oxide) increased the carrier fluid’s thermal properties considerably. It is more effective at dealing with low energy transmission.

## 1. Introduction

Fluid flow over the substrate of a rotating disc has garnered considerable attention because of its consequences in real phenomena. Flow over the rotating disc surface is commonly used in electric power generation systems, co rotating machines, aerodynamics engineering, rotating equipment, geothermal sector, chemical reaction, and computer processing [1]. Accurate analysis and precise measurements of reaction are the prerequisite for better understanding the factors that overcome kinetics of reaction. Electrode system of rotating disk is broadly used to examine the reactions kinetics and that are suffering from mass transmission deficiency [2]. Tassaddiq *et al*. [3] examined the MHD, an incompressible iron ferrite and carbon nanotubes CNTs hybrid nanoliquid over an impermeable spinning disk. They perceived that the fluid temperature and velocity are considerably increased by increasing disc rotating velocity. Mustafa *et al*. [4] used the Bingham fluid model to analyze the heat transmission and viscous dissipation for swirling viscoelastic fluid flow crossing over a permeable spinning disc. Using a greater suction velocity at the disc allows for a faster thermal efficiency. However, as the fluid yield stress increases, the heat transfer is expected to deteriorate. Hafeez and Khan [5] scrutinized the mass and heat flux theories in Oldroyd-B fluid flow over an extending disk. It should be noted that the solutal relaxation time coefficient has a negative impact on mass transmission rate. Gul *et al*. [6] modeled the hybrid nanofluid flow between disk and cone with several cases. Based on the analysis of the results, it was concluded that a rotating disc that a rotating disc with a fixed cone would attain the intended cooling of disk-cone tools if the surface temperature remains steady.

Fluid that is commonly used Ethanol, kerosene oil and water etc., which play a prominent role in energy transfer, heating and air conditioning processes, energy generation, and other small electronics mechanisms. However, since these liquids have a poor thermal energy transfer capacity, they are unable to accommodate the need for high heat exchange rates. Nanometer-sized particles (1-100nm), also recognized as nanomaterials, are applied to a natural fluid to substitute for this inadequacy and increase thermal conductivity. To quantify the thermophysical properties of various kinds of nanoparticles, the researcher used a variety of techniques [7]. Nanoparticles are used to clean surfaces in engineering because they have the ability to disperse and wet oil. It improves thermal efficiency, which is important in the fields of energy production, hypoxia, micro fabrication and metallurgy. Magnesium oxide *MgO* is a compound made up of *Mg*^2+^ and *O*^2-^ ions that are bonded together through a powerful ionic bond. It is made by calcination of *Mg (OH)* ^2^ and *MgCO*^3^ at temperature range from 700°C-1500°C. Most of it is utilized in the hematologic and electrical industries. Similarly, *Ag* particles with antibacterial properties are used to control microorganism growth in wound, burns and surgical apparatus and infrastructure. Microorganisms are particularly toxic to compound based on silver and its ions. Hussanan *et al*. [8] investigated oxide nanomaterials in engine nanoliquids and water for energy up gradation. Using the finite element technique, Ghalambaz *et al*. [9] investigated convection flow of a hybrid nanofluid (Ag–MgO/water) within a cavity. Copper-Alumina micro materials were listed for the base fluid. Aluminum Oxide was used by Motlagh and Soltanpour [10] to elaborate the heat transition in a non-flat cavity. Acharya *et al*. [11] investigated the hybrid fluid flow over a spinning disc with the consequences of MHD and Hall current. They introduced the *TiO*_*2*_ and *Cu* nanomaterials to creative a class of nanoliquid. The magnetic interaction on flow with CNTs nano powders via a porous tube is studied by Akber *et al*. [12]. Shah *et al*. [13] reported that an angled magnetic field, performance of copper selenide and the efficacy of chemical catalytic reactors effects the energy and concentration distribution of nanofluids. Sowmya *et al*. [14] uncovered natural convective thermal transference in a rectangular chamber with two heated fins on the bottom wall for a hybrid nanofluid flow employing iron oxide and silver nanomaterials. Zhou *et al*. [15] Khan addressed Von Karman’s classic swirling flow for Maxwell fluid over a porous whirling disc with a constant suction/injection mechanism.

In the engineering sectors, complex nonlinear boundary value problems that cannot be addressed usually are often observed. Convergence is sensitive to the relaxation parameters and initial approach for many problems that are commonly solved by other numerical algorithms. The PCM’s goal is to explore the method’s generalizability as a feasible alternative to nonlinear problems [16]. Shuaib *et al*. [17] emphasized the 3D unsteady fluid and heat propagation across the substrate of a non-flat stretchable rotating disc. The fluid has investigated under the effects of a magnetic strength from the outside. The feature of an ionic transition boundary layer flow over a gyrating disc was discovered by Shuaib *et al*. [18]. The Poisson’s, Nernst-Planck equation and Navier Stokes equations, were used to solve for the ionic compounds. Wang *et al*. [19] presented stability analysis of nonlinear problems for engineering applications through parametric continuation algorithm. They also looked at the static bifurcation that happens when solving nonlinear initial value problems with different characteristic roots and provided an algorithm for instantly calculating the bifurcation points.

The aim of this study is to modify an idea of Ref. [20,21], by investigating the influence of two separate nanoparticles, Silver *Ag/*water and magnesium oxide *MgO*/water hybrid nanofluids, on a wavy spinning disc that moves upward and downward. This research is being considered in order to increase the thermal conductivity of the fluid flow. The modelled equations are solved numerically using the parametric continuation process (PCM), and the results have been validated and compared with Matlab’s package boundary value solver (bvp4c). All outcomes are in the closest possible agreement with one another.

## 2. Mathematical formulation

The physical analysis of the problem, physicochemical characteristics and the equation of motion has been discussed in this section.

### 2.1 Physical description of the problem

We have assumed a 3D flow of *Ag-MgO*/Water (Silver and magnesium-oxide) hybrid nanoliquid over a wavy up and down moving spinning disk. Initially the disk is at *a*(0) = *h*. At a vertical distance *Z* = *a*(*t*) the disk is moving with velocity *ω* = *a*(*t*). The consequences of buoyancy impacts are negligible. The disk is revolving about *z*-axis with angular velocity Ω(*t*), and it has been considered that the *Ag-MgO* nano-size particles are scattered consistent. The magnetic field of constant magnitude is uniformly applied with $\vec{B}=(B_{r}{\vec{e}}_{r}+B_{\theta }{\vec{e}}_{\theta })$ and $B=\sqrt{B_{r}^{2}+B_{\theta }^{2}}$ respectively, where ${\vec{e}}_{r}$ and ${\vec{e}}_{\theta }$ are unit vectors.

### 2.2 Equation of motion

The modeled equations, based on above presumption are expressed as [20,21]:

$$\frac{\partial u}{\partial r}+\frac{\partial w}{\partial z}+\frac{u}{r}=0,$$ (1)

$$\rho_{hnf}(\frac{\partial u}{\partial t}+u\frac{\partial u}{\partial r}+w\frac{\partial u}{\partial z}-\frac{v^{2}}{r})=-\frac{\partial p}{\partial r}+\mu_{hnf}(\frac{\partial^{2}u}{\partial r^{2}}+\frac{\partial^{2}u}{\partial z^{2}}+\frac{1}{r}\frac{\partial u}{\partial r}-\frac{u}{r^{2}})+F_{r},$$ (2)

$$\rho_{hnf}(\frac{\partial v}{\partial t}+u\frac{\partial v}{\partial r}+w\frac{\partial v}{\partial z}-\frac{uv}{r})=\mu_{hnf}(\frac{\partial^{2}v}{\partial r^{2}}+\frac{\partial^{2}v}{\partial z^{2}}+\frac{1}{r}\frac{\partial v}{\partial r}-\frac{v}{r^{2}}),$$ (3)

$$\rho_{hnf}(\frac{\partial w}{\partial t}+u\frac{\partial w}{\partial r}+w\frac{\partial w}{\partial z})=-\frac{\partial p}{\partial r}+\mu_{hnf}(\frac{\partial^{2}w}{\partial r^{2}}+\frac{\partial^{2}w}{\partial z^{2}}+\frac{1}{r}\frac{\partial w}{\partial r})+F_{\theta },$$ (4)

$$(\frac{\partial T}{\partial t}+u\frac{\partial T}{\partial r}+w\frac{\partial T}{\partial z})=\frac{k}{{\left(\rho C_{p}\right)}_{hnf}}(\frac{\partial^{2}T}{\partial r^{2}}+\frac{1}{r}\frac{\partial T}{\partial r}+\frac{\partial^{2}T}{\partial z^{2}}),$$ (5)

$$(\frac{\partial B_{r}}{\partial t})=-w\frac{\partial B_{r}}{\partial z}-Br\frac{\partial B_{w}}{\partial z}+u\frac{\partial B_{z}}{\partial z}+Bz\frac{\partial u}{\partial z}+\frac{1}{\sigma \mu }(\frac{\partial^{2}B_{r}}{\partial r^{2}}+\frac{\partial^{2}B_{r}}{\partial z^{2}}+\frac{1}{r}\frac{\partial B_{r}}{\partial r}-\frac{Br}{r^{2}}),$$ (6)

$$(\frac{\partial B_{z}}{\partial t})=w\frac{\partial B_{r}}{\partial r}+Br\frac{\partial B_{w}}{\partial r}+\frac{1}{r}wBr-u\frac{\partial B_{z}}{\partial r}-Bz\frac{\partial u}{\partial r}-\frac{1}{r}uBz+\frac{1}{\sigma \mu }(\frac{\partial^{2}B_{z}}{\partial r^{2}}+\frac{\partial^{2}B_{z}}{\partial z^{2}}+\frac{1}{r}\frac{\partial B_{z}}{\partial r}),$$ (7)

Here *F*_*r*_ and *F*_*θ*_ along *x* and *z* direction are the body forces, respectively, which can be stated as [21]:

$$F_{r}=\frac{Ha^{2}\mu_{hnf}}{R^{2}}(v\sin\theta \cos\theta -u\sin^{2}\theta ),$$ (8)

$$F_{\theta }=\frac{Ha^{2}\mu_{hnf}}{R^{2}}(u\sin\theta \cos\theta -v\sin^{2}\theta ),$$ (9)

Here, Ha is $LB_{0}\sqrt{\frac{\sigma }{\mu }}$, in which *θ* is the direction and *B*_0_ is the magnitude of magnetic field, where the velocity components are specified as *u*,*v*,*w*.

### 2.3 Boundary condition

The initial and boundary conditions for wavy gyrating disk are:

$$u=0,\;v=r\Omega_{0}(t),\;w=w_{0}(t),\;T=T_{0},\;Br=0,\;Bz=0\;at\;z=0$$

$$u\to 0,\;v\to 0,\;w\to 0,\;T\to T_{\infty },\;Br=\frac{M_{0}}{2R},Bz=-\alpha M_{0}\;at\;z\to \infty .$$ (10)

### 2.4 Thermophysical properties of nanoliquid

The density and specific heat capacity of the hybrid nanofluid can be revealed as [21]:

$$\rho_{hnf}=\{\left(1-\varphi_{2}\right)[{\left(1-\varphi_{1}\right)}_{\rho_{f}}+\varphi_{1}\rho_{s1}]\}+\varphi_{2}\rho_{s2},$$ (11)

$${\left(\rho C_{p}\right)}_{hnf}=\{\left(1-\varphi_{2}\right)[\left(1-\varphi_{1}\right){\left(\rho C_{p}\right)}_{f}+\varphi_{1}{\left(\rho C_{p}\right)}_{s1}]\}+\varphi_{2}{\left(\rho C_{p}\right)}_{s2},$$ (12)

where (*C*_*p*_)_*s*1_,(*C*_*p*_)_*s*2_ are specific heat capacity, *ρ*_*s*1_,*ρ*_*s*2_ are the density and *φ*_1_,*φ*_2_ are the volume friction of the *Ag-MgO* hybrid nanoliquid respectively shown in Table 1.

**Table 1:** The numerical properties of water and hybrid nanofluid [20].

	*ρ*(kg/m^3^)	*C*_*p*_(j/kgK)	*k*(W/mK)	*β*×10^5^(*K*^−1^)
Pure water	997.1	4179	0.613	21
Magnesium oxide	3560	955	45	1.80
Silver	10,500	235	429	1.89

The hybrid nanofluid viscosity *μ*_*hnf*_ is expressed as [22]:

$$\frac{\mu_{hnf}}{\mu_{bf}}=\frac{1}{{\left(1-\varphi_{1}\right)}^{2.5}{\left(1-\varphi_{2}\right)}^{2.5}},$$ (13)

Here, the thermal conductivity and Prandtl number is expressed as [21]:

$$k_{hnf}=-\frac{q_{w}}{\partial \theta /\partial y},\;{\mathrm{Pr}}_{hnf}=\frac{{\left(\mu C_{p}\right)}_{hnf}}{k_{hnf}}.$$ (14)

### 2.5 Karman’s approach

We use the following similarity framework, to reduce Eqs (1)–(7) and (10) to the system of ODEs [20]:

$$u=\frac{rv}{a^{2}(t)}f(\eta ),\;v=\frac{rv}{a^{2}(t)}g(\eta ),\;w=\frac{v}{a(t)}h(\eta ),\;p=\frac{pv^{2}}{a^{2}(t)}p(\eta ),\;Br=\frac{r\Omega M_{0}}{a(t)}m^{\prime }(\eta ),$$

$$Bz=\frac{M_{0}{\left(2\nu_{f}\Omega \right)}^{1/2}}{a(t)}n(\eta ),\;T=T_{\infty }+\Delta T_{\infty },\;\eta =\frac{Z}{a(t)}-1,\;\eta_{Z}=\frac{1}{a(t)},\;\eta_{t}=\frac{-a(t)}{a(t)}(\eta +1).$$ (15)

As a result of Eq (15), we get the following:

$$f^{\prime \prime }=\frac{\rho_{hnf}}{\mu_{hnf}}(hf^{\prime }+f^{2}-g^{2}-S\frac{(\eta +1)f^{\prime }}{2}+f)+A\omega (g\sin\theta \cos\theta -f\sin^{2}\theta ),$$ (16)

$$g^{\prime \prime }=\frac{\rho_{hnf}}{\mu_{hnf}}(hg^{\prime }+2fg-S(\frac{(\eta +1)g^{\prime }}{2}-g)),$$ (17)

$$h^{\prime \prime }=\frac{\rho_{hnf}}{\mu_{hnf}}(hh^{\prime }-S\frac{(\eta +1)h^{\prime }}{2}+h^{\prime })-A\omega (f\sin\theta \cos\theta -g\sin^{2}\theta ),$$ (18)

$$\theta^{\prime \prime }=\rho_{hnf}(h\theta^{\prime }-S(\frac{(\eta +1)\theta^{\prime }}{2}+\gamma \theta )),$$ (19)

$$m^{\prime \prime \prime }=Bt(-hm^{\prime \prime }+m^{\prime }h^{\prime }+fn^{\prime }+nf^{\prime }+S(\frac{m^{\prime \prime }\eta }{2}+m^{\prime })),$$ (20)

$$n^{\prime \prime }=-Bt(2hm^{\prime }+2nf-\frac{S}{2}(n^{\prime }\eta +n)).$$ (21)

The transform conditions are:

$$f(0)=0,\;h(0)=\beta \frac{S}{2},\;g(0)=\omega ,\theta (0)=m^{\prime }(0)=n(0)=1\;\mathrm{at}\;\eta =0,$$

$$f(\eta )\to 0,\;g(\eta )\to 0,\;h(\eta )\to 0,\;\theta (\eta )\to 0,\;m^{\prime }(\eta )\to 0,n(\eta )\to 0,\;\mathrm{as}\;\eta =\infty ,$$ (22)

Sign *ω* edify disk’s rotation, *S* controlled the up and down fluctuating of the disk and *γ* is the is the thermal energy parameter, which can be defined as [18]:

$$S=2\frac{a^{*}(t)a(t)}{v},\;\omega =2\frac{a^{2}(t)\Omega (t)}{v},\;\gamma =\frac{1}{2}\frac{a(t)T}{a^{*}(t)\Delta T}.$$ (23)

The dimensionless form of skin friction and Nusselt number is:

$$C_{f}=\frac{\sqrt{\tau_{wr}^{2}-\tau_{w\phi }^{2}}}{\rho_{f}{\left(\Omega r\right)}^{2}}\;\mathrm{and}\;Nu=\frac{rq_{w}}{k_{f}(T_{w}-T_{\infty })}.$$ (24)

Where, *τ*_*wr*_ and *τ*_*wϕ*_ represent the radial and transverse stress respectively.

### 3. Numerical solution

The following steps have been used, while solving the system of ODE (16–21) and their boundary conditions (22):

**Step 1: Reducing the system of modeled equations to the first order ODE** we introduced the following similarity variables:

$$\begin{array}{l} \chi_{1}(\eta )=f(\eta ),\;\chi_{2}(\eta )=f^{\prime }(\eta ),\;\chi_{3}(\eta )=g(\eta ),\;\\ \chi_{4}(\eta )=g^{\prime }(\eta ),\;\chi_{5}(\eta )=h(\eta ),\;\chi_{6}(\eta )=h^{\prime }(\eta ),\;\\ \chi_{7}(\eta )=\theta (\eta ),\;\chi_{8}(\eta )=\theta^{\prime }(\eta ),\;\chi_{9}(\eta )=m(\eta ),\;\\ \chi_{10}(\eta )=m^{\prime }(\eta ),\;\chi_{11}(\eta )=m^{\prime \prime }(\eta ),\;\chi_{12}(\eta )=n(\eta ),\;\\ \;\chi_{13}(\eta )=n^{\prime }(\eta ), \end{array})$$ (25)

Using Eq (25) into the BVP (16–21) and (22), we get:

$$\begin{array}{l} \chi_{2}{}^{\prime }(\eta )=\frac{\rho_{hnf}}{\mu_{hnf}}\left((\chi_{5}(\eta )-S\frac{\left(\eta +1\right)}{2})\chi_{2}(\eta )+{\left(\chi_{1}(\eta )\right)}^{2}-{\left(\chi_{3}(\eta )\right)}^{2}-S\chi_{1}(\eta )\right)\\ +A\omega (\chi_{3}(\eta )\sin\theta \cos\theta -\chi_{1}(\eta )\sin^{2}\theta ) \end{array}$$ (26)

$$\chi_{4}{}^{\prime }(\eta )=\frac{\rho_{hnf}}{\mu_{hnf}}(\left(\chi_{5}(\eta )-S\frac{\left(\eta +1\right)}{2})\chi_{4}(\eta )+2\chi_{1}(\eta )\chi_{3}(\eta )-S\chi_{3}(\eta \right))$$ (27)

$$\chi_{6}{}^{\prime }(\eta )=\frac{\rho_{hnf}}{\mu_{hnf}}(\left(\chi_{5}(\eta )-S\frac{\left(\eta +1\right)}{2}+1)\chi_{6}(\eta \right))-A\omega \left(\chi_{1}(\eta )\sin\theta \cos\theta -\chi_{3}(\eta )\sin^{2}\theta \right)$$ (28)

$$\chi_{8}{}^{\prime }(\eta )=\rho_{hnf}(\left(\chi_{5}(\eta )-S\frac{\left(\eta +1\right)}{2})\chi_{8}(\eta )-S\chi_{7}\gamma (\eta \right))$$ (29)

$$\chi_{11}{}^{\prime }(\eta )=Bt(\left(-\chi_{5}(\eta )-\frac{S\eta }{2})\chi_{11}(\eta )+S\chi_{10}(\eta )+\chi_{10}(\eta )\chi_{6}(\eta )+\chi_{1}(\eta )\chi_{13}(\eta )+\chi_{13}(\eta )\chi_{2}(\eta \right))$$ (30)

$$\chi_{13}{}^{\prime }(\eta )=-Bt(-\frac{S}{2}(\eta \chi_{13}(\eta )+\chi_{12}(\eta ))2\chi_{1}(\eta )\chi_{12}(\eta )+2\chi_{5}(\eta )\chi_{10}(\eta ))$$ (31)

the boundary conditions are:

$$\begin{array}{l} \chi_{1}(\eta )=0,\;\chi_{3}(\eta )=\omega ,\;\chi_{5}(\eta )=\beta \frac{S}{2},\;\chi_{7}(\eta )=\chi_{10}(\eta )=\chi_{12}(\eta )=1\;at\;\eta =0,\;\\ \chi_{1}(\eta )\to 0,\;\chi_{3}(\eta )\to 0,\;\chi_{5}(\eta )\to 0,\;\chi_{7}(\eta )\to 0,\;\chi_{10}(\eta )\to 0,\;\chi_{12}(\eta )\to 0\;at\;\eta \to \infty . \end{array}$$ (32)

**Step 2**: **By introducing the embedding parameter p**

We introduced the parameter p in the above system of Eqs (26–31) as follow:

$$\begin{array}{l} \chi_{2}{}^{\prime }(\eta )=\frac{\rho_{hnf}}{\mu_{hnf}}\left((\chi_{5}(\eta )-S\frac{\left(\eta +1\right)}{2})(\chi_{2}(\eta )-1)p+{\left(\chi_{1}(\eta )\right)}^{2}-{\left(\chi_{3}(\eta )\right)}^{2}-S\chi_{1}(\eta )\right)\\ +A\omega (\chi_{3}(\eta )\sin\theta \cos\theta -\chi_{1}(\eta )\sin^{2}\theta ) \end{array}$$ (33)

$$\chi_{4}{}^{\prime }(\eta )=\frac{\rho_{hnf}}{\mu_{hnf}}(\left(\chi_{5}(\eta )-S\frac{\left(\eta +1\right)}{2})(\chi_{4}(\eta )-1)p+2\chi_{1}(\eta )\chi_{3}(\eta )-S\chi_{3}(\eta \right))$$ (34)

$$\chi_{6}{}^{\prime }(\eta )=\frac{\rho_{hnf}}{\mu_{hnf}}((\chi_{5}(\eta )-S\frac{\left(\eta +1\right)}{2}+1)(\chi_{6}(\eta )-1)p)-A\omega (\chi_{1}(\eta )\sin\theta \cos\theta -\chi_{3}(\eta )\sin^{2}\theta )$$ (35)

$$\chi_{8}{}^{\prime }(\eta )=\rho_{hnf}(\left(\chi_{5}(\eta )-S\frac{\left(\eta +1\right)}{2})(\chi_{8}(\eta )-1)p-S\chi_{7}\gamma (\eta \right))$$ (36)

$$\begin{array}{l} \chi_{11}{}^{\prime }(\eta )=Bt\left((-\chi_{5}(\eta )-\frac{S\eta }{2})(\chi_{11}(\eta )-1)p+S\chi_{10}(\eta )+\chi_{10}(\eta )\chi_{6}(\eta )\right)\\ +(+\chi_{1}(\eta )\chi_{13}(\eta )+\chi_{13}(\eta )\chi_{2}(\eta )) \end{array}$$ (37)

$$\chi_{13}{}^{\prime }(\eta )=-Bt(-\frac{S}{2}(\eta \chi_{13}(\eta )+(\chi_{12}(\eta )-1)p)2\chi_{1}(\eta )\chi_{12}(\eta )+2\chi_{5}(\eta )\chi_{10}(\eta ))$$ (38)

**Step 3**: **Differentiating w.r.t parameter ‘p’**

Eqs (33–38) after differentiating w.r.t parameter p reform to the following structure with respect to parameter p

$$V^{\prime }=AV+R,$$ (39)

where, R is the remainder and A is the coefficient matrix:

$$\frac{d\chi_{i}}{d\tau }$$ (40)

where *i* = 1, 2, *………*11.

**Step 4: Apply specify Cauchy problem and superposition principle for each component**

V=aU+W, (41)

where U, W are unknown vector functions and *a* is unknown blend coefficient. Solving the Cauchy problems for each component

$$U^{\prime }=aU,$$ (42)

$$W^{\prime }=AW+R,$$ (43)

by putting Eq (41) into the original Eq (39), we get

$$(aU+W{)}^{\prime }=A(aU+W)+R,$$ (44)

Step **5: Solving the Cauchy problems**

The numerical implicit scheme is implemented in the proposed problem, presented as follow: From Eqs (42) and (43)

$$\frac{U^{i+1}-U^{i}}{\Delta \eta }=AU^{i+1},\;or\;(I-\Delta \eta A)U^{i+1}=U^{i},$$ (45)

$$\frac{W^{i+1}-W^{i}}{\Delta \eta }=AW^{i+1},\;or\;(I-\Delta \eta A)W^{i+1}=W^{i},$$ (46)

here, we obtained the iterative form of the problem.


$$U^{i+1}={\left(I-\Delta \eta A\right)}^{-1}U^{i},$$ (47)

$$W^{i+1}={\left(I-\Delta \eta A\right)}^{-1}(W^{i}+\Delta \eta R).$$ (48)

## 4. Analysis and discussion of results

### 4.1 Analysis

**Fig 1:**
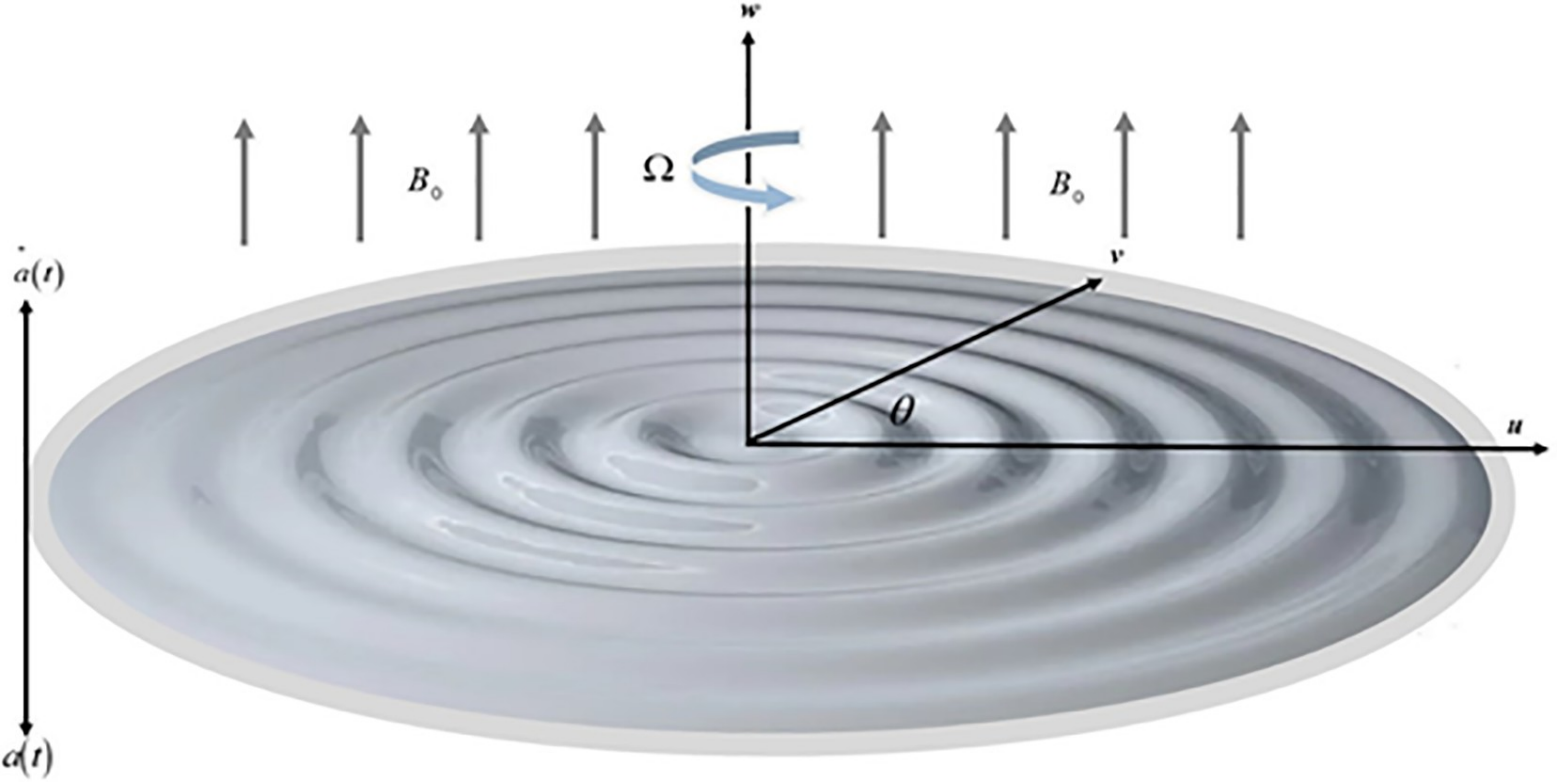


**Fig 2:**
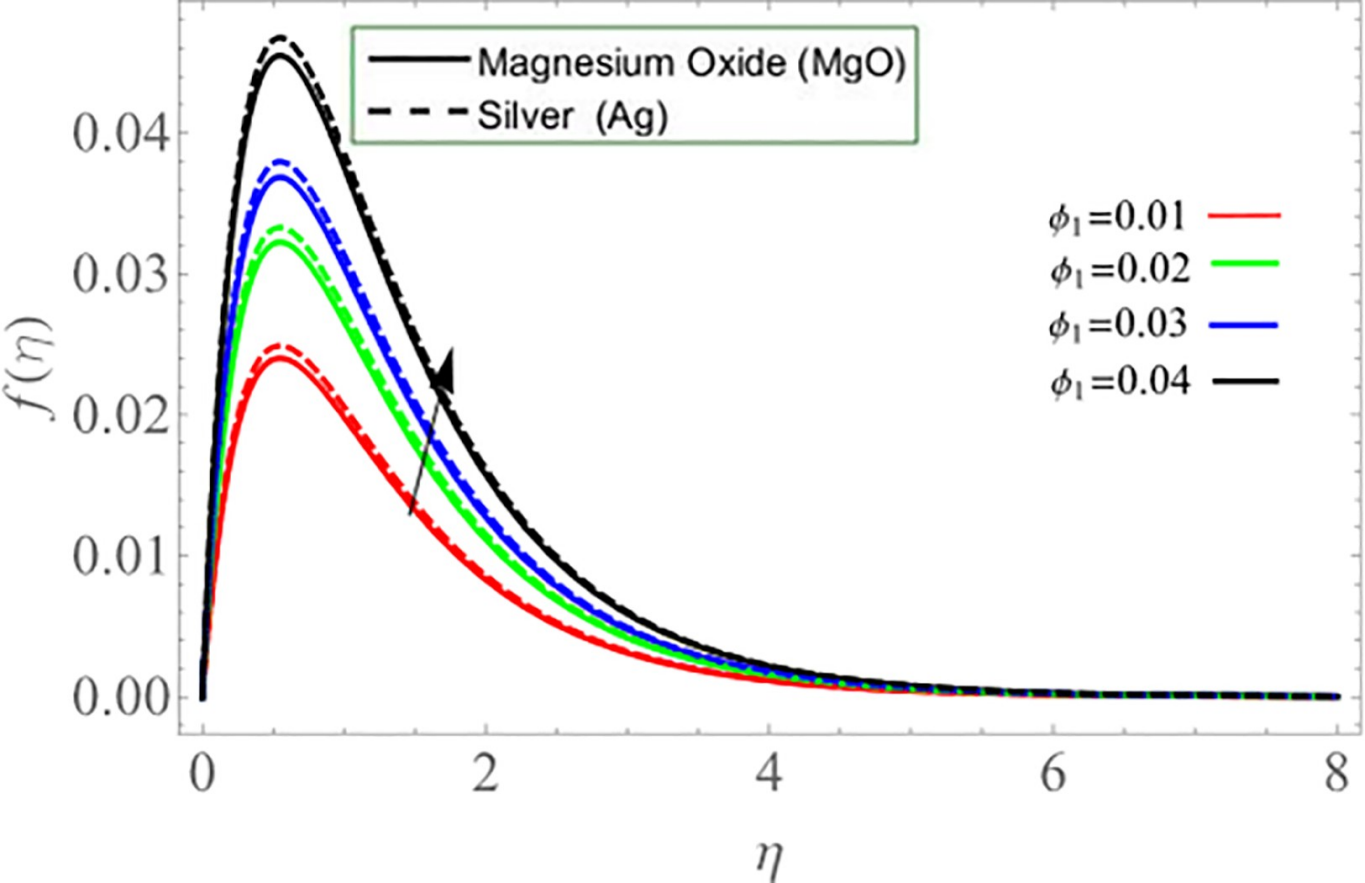


**Fig 3:**
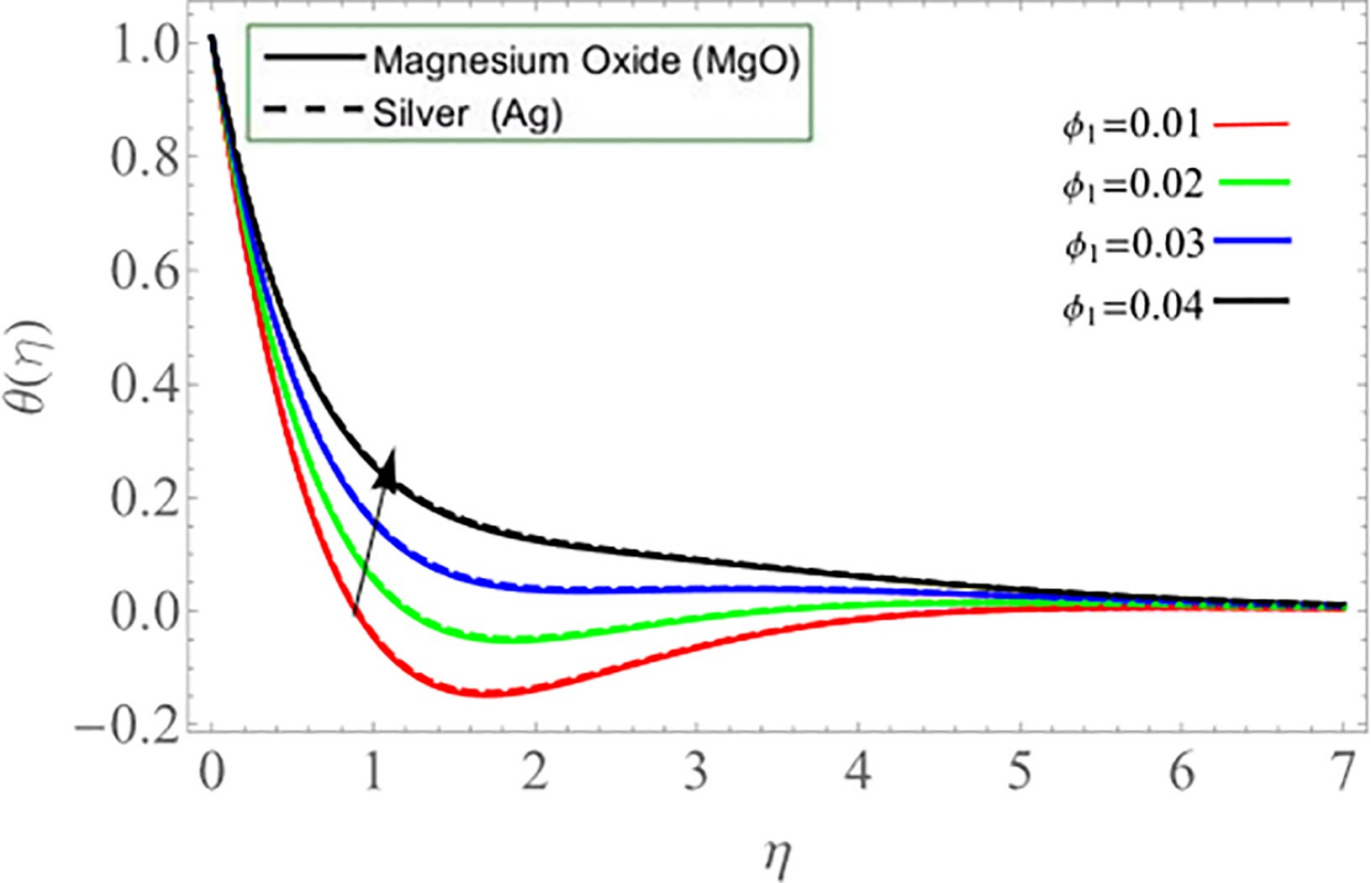


**Fig 4:**
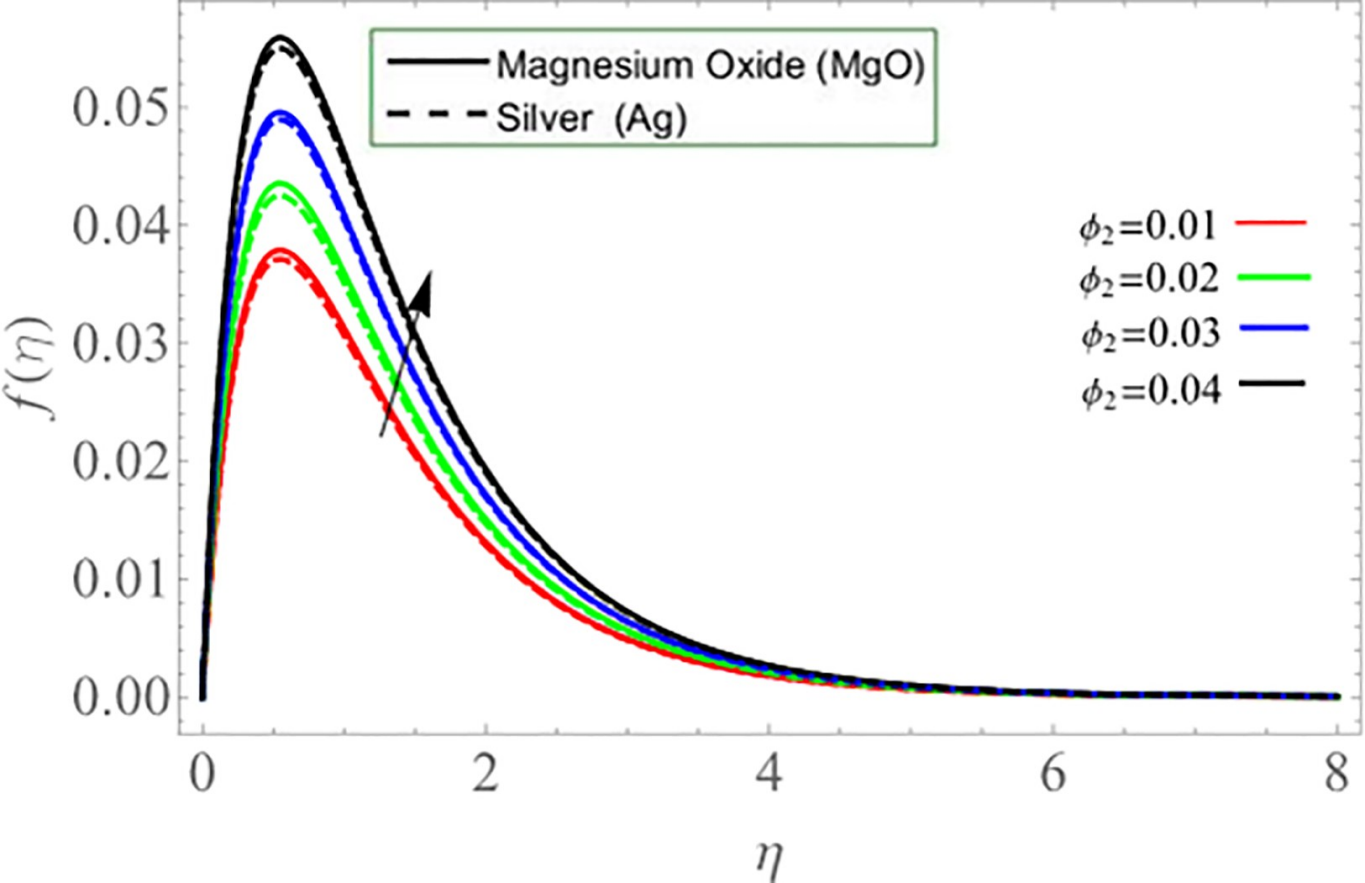


**Fig 5:**
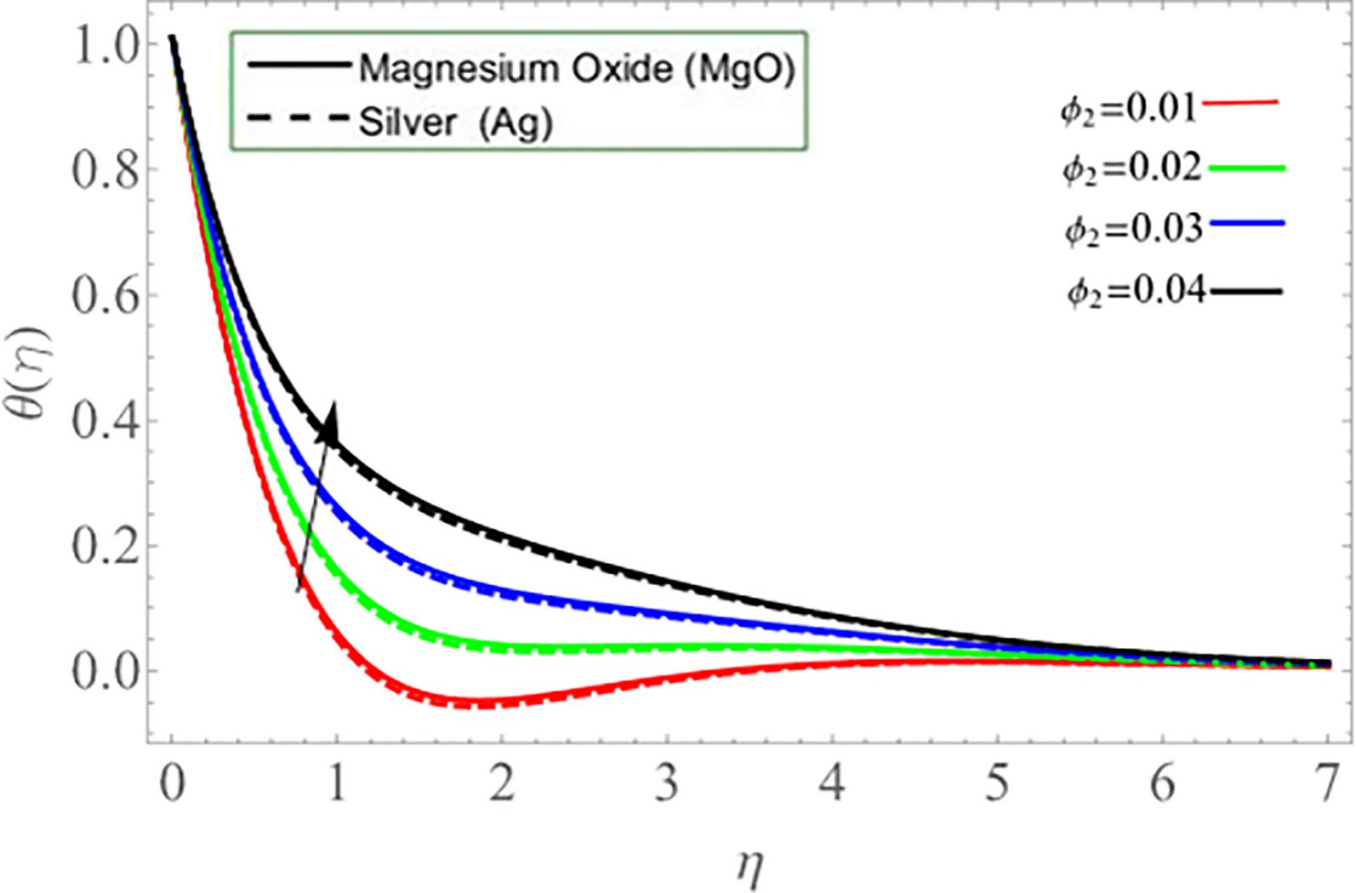


**Fig 6:**
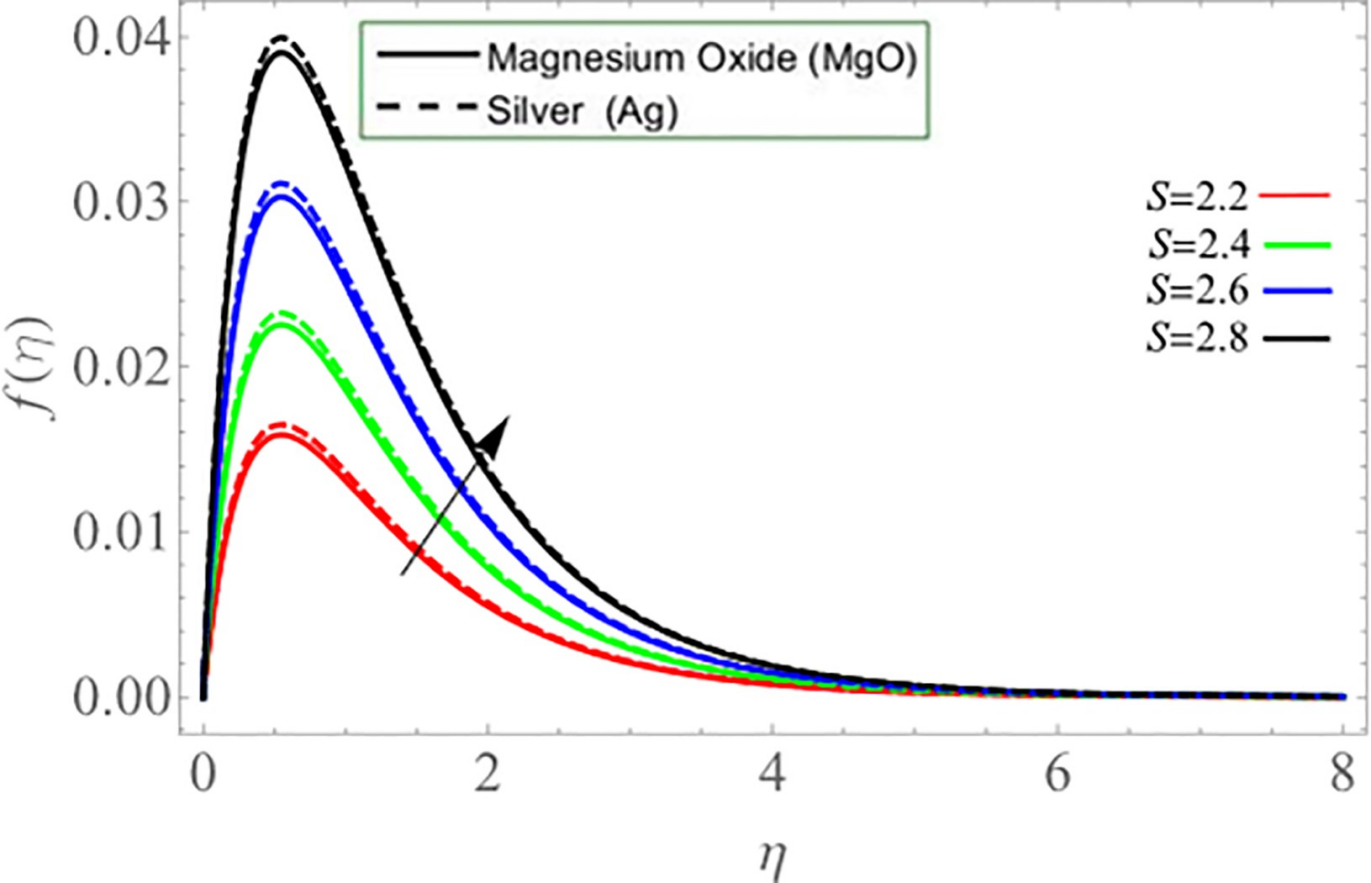


**Fig 7:**
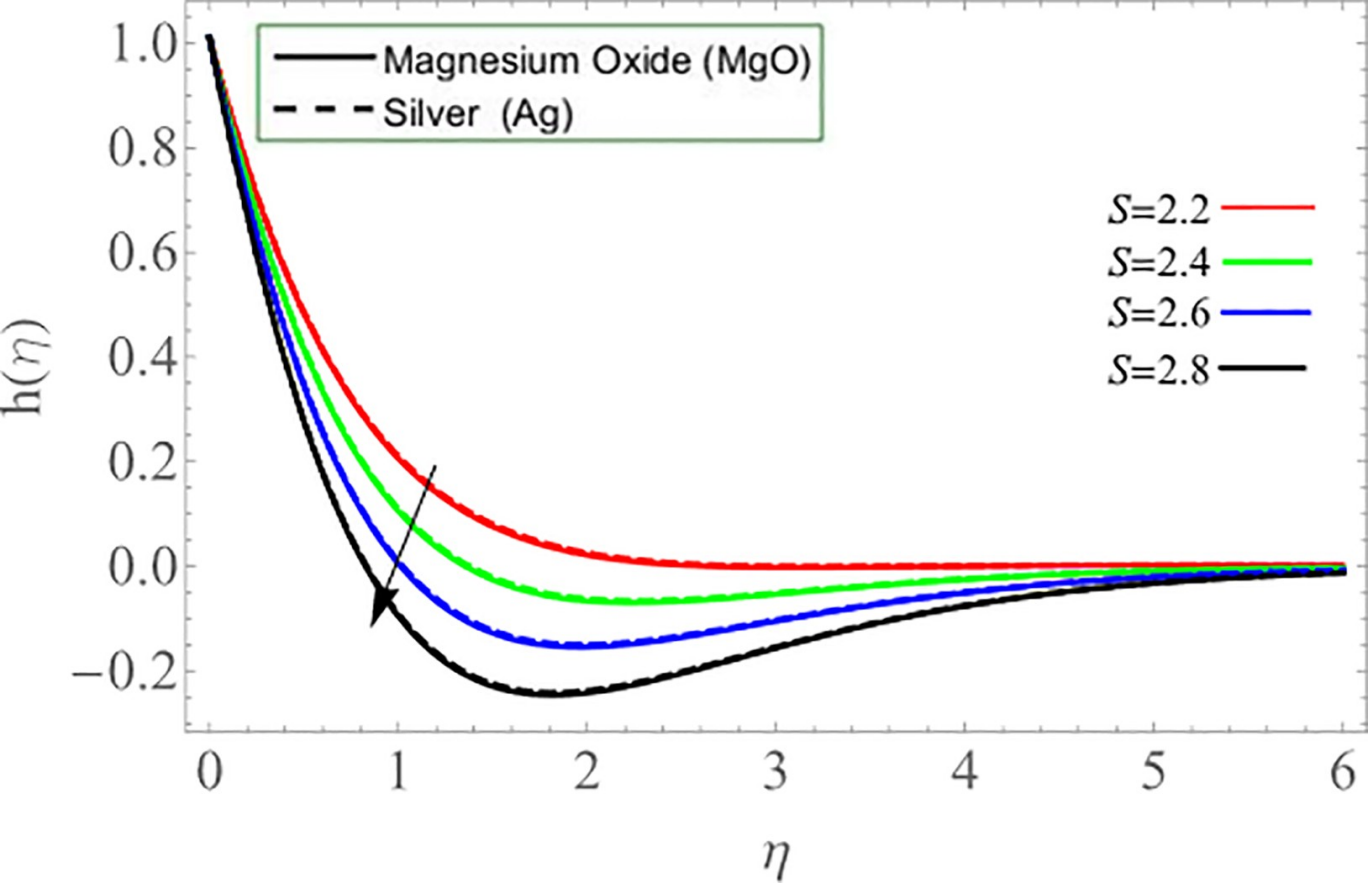


**Fig 8:**
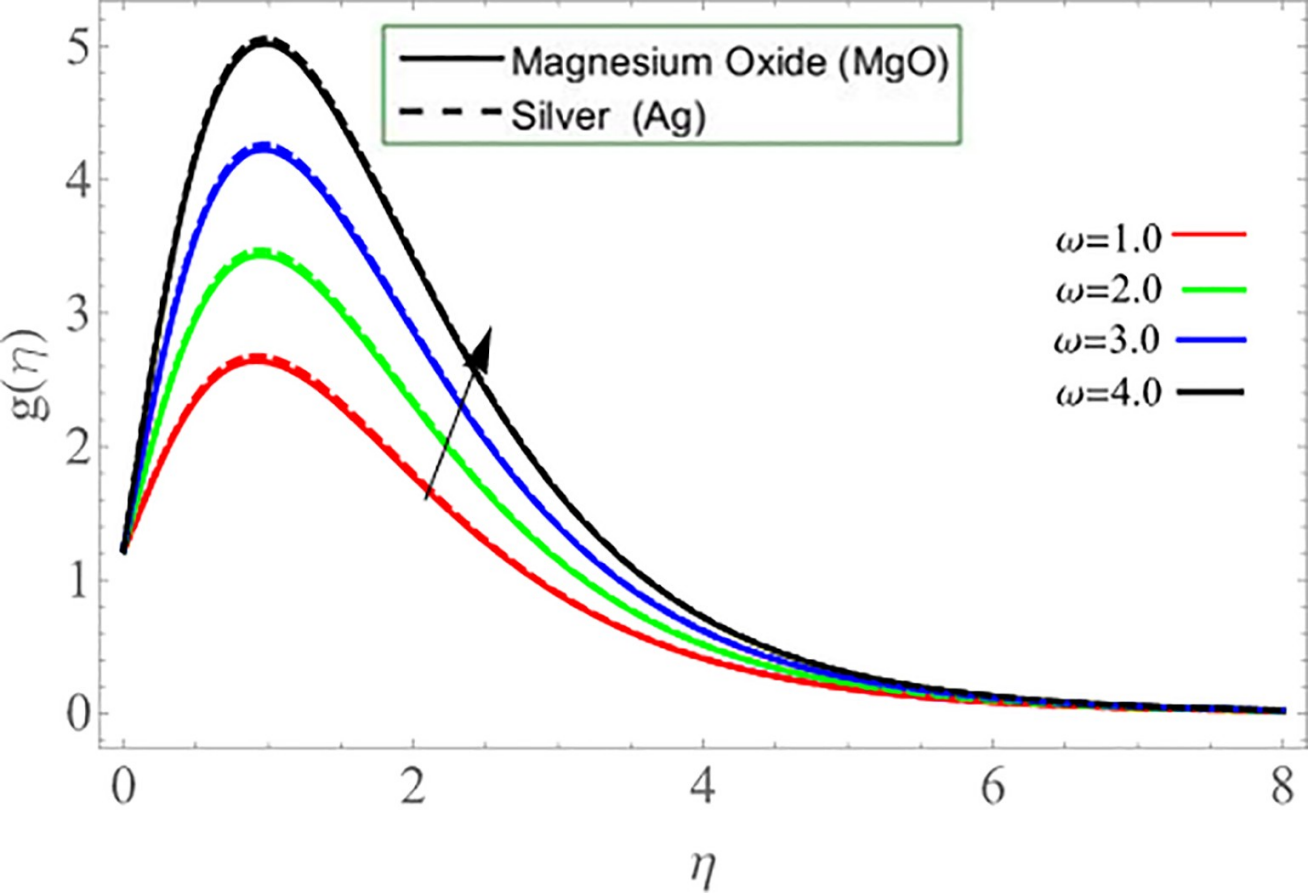


**Fig 9:**
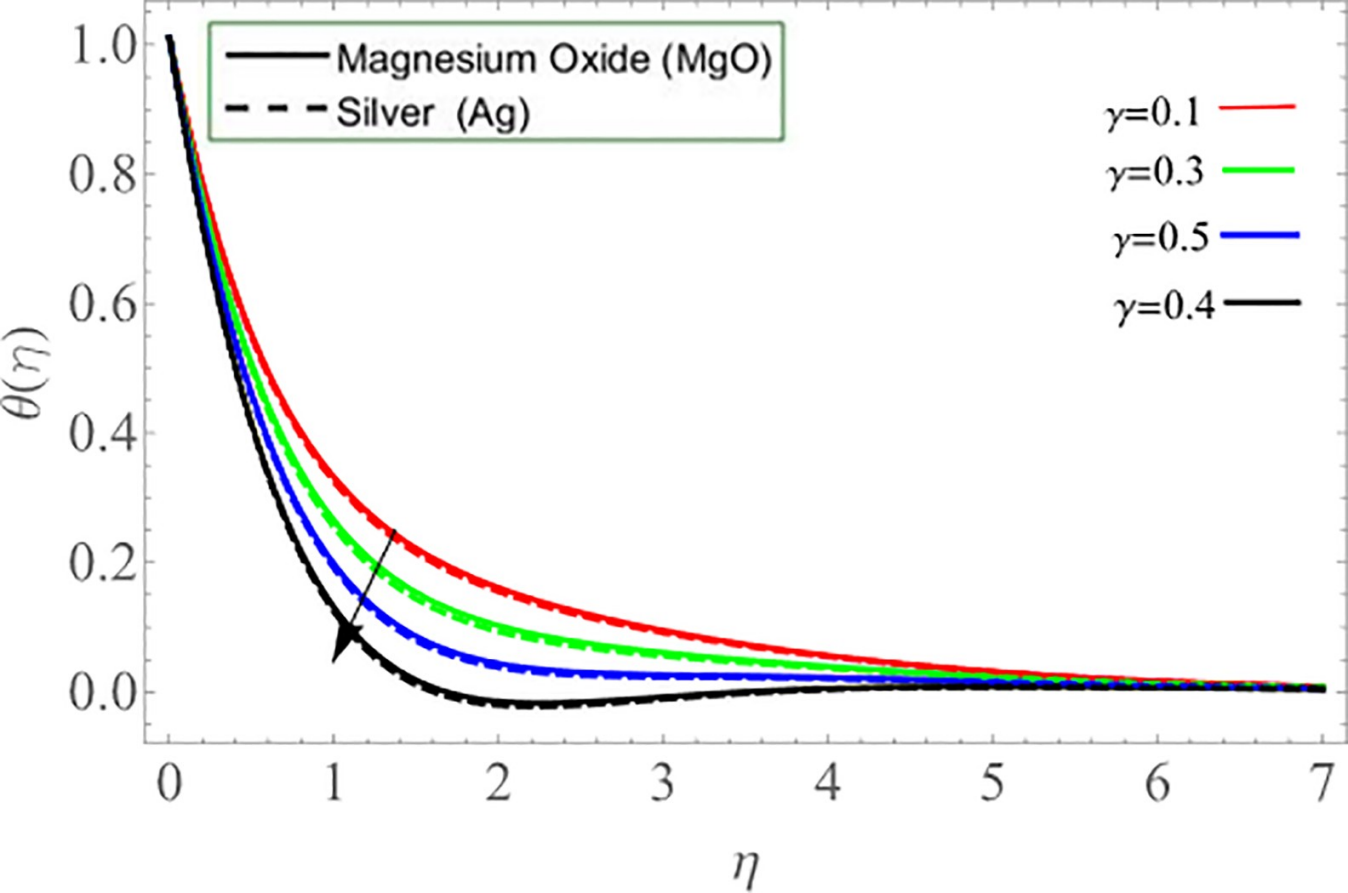


**Fig 10:**
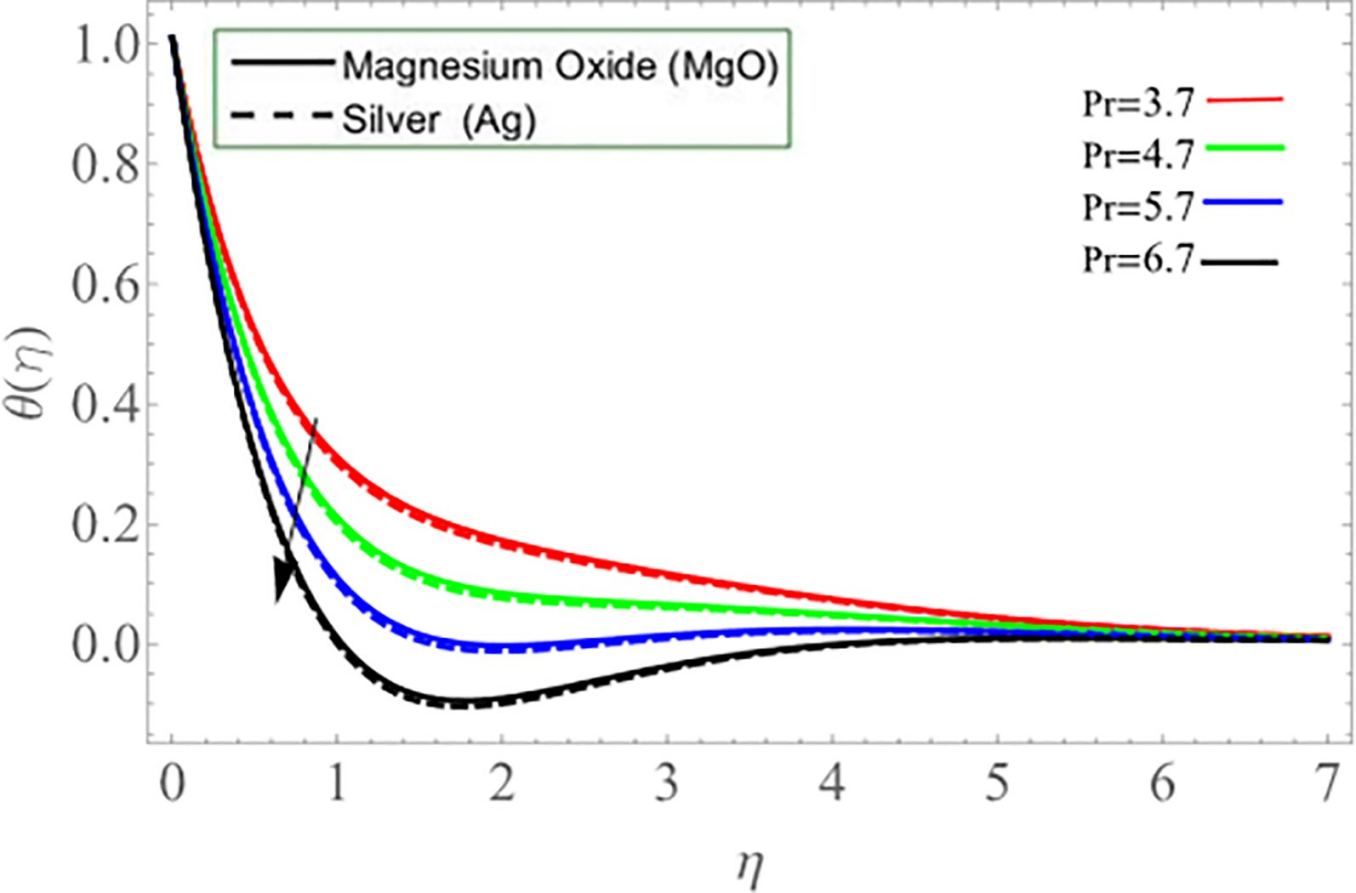


**Fig 11:**
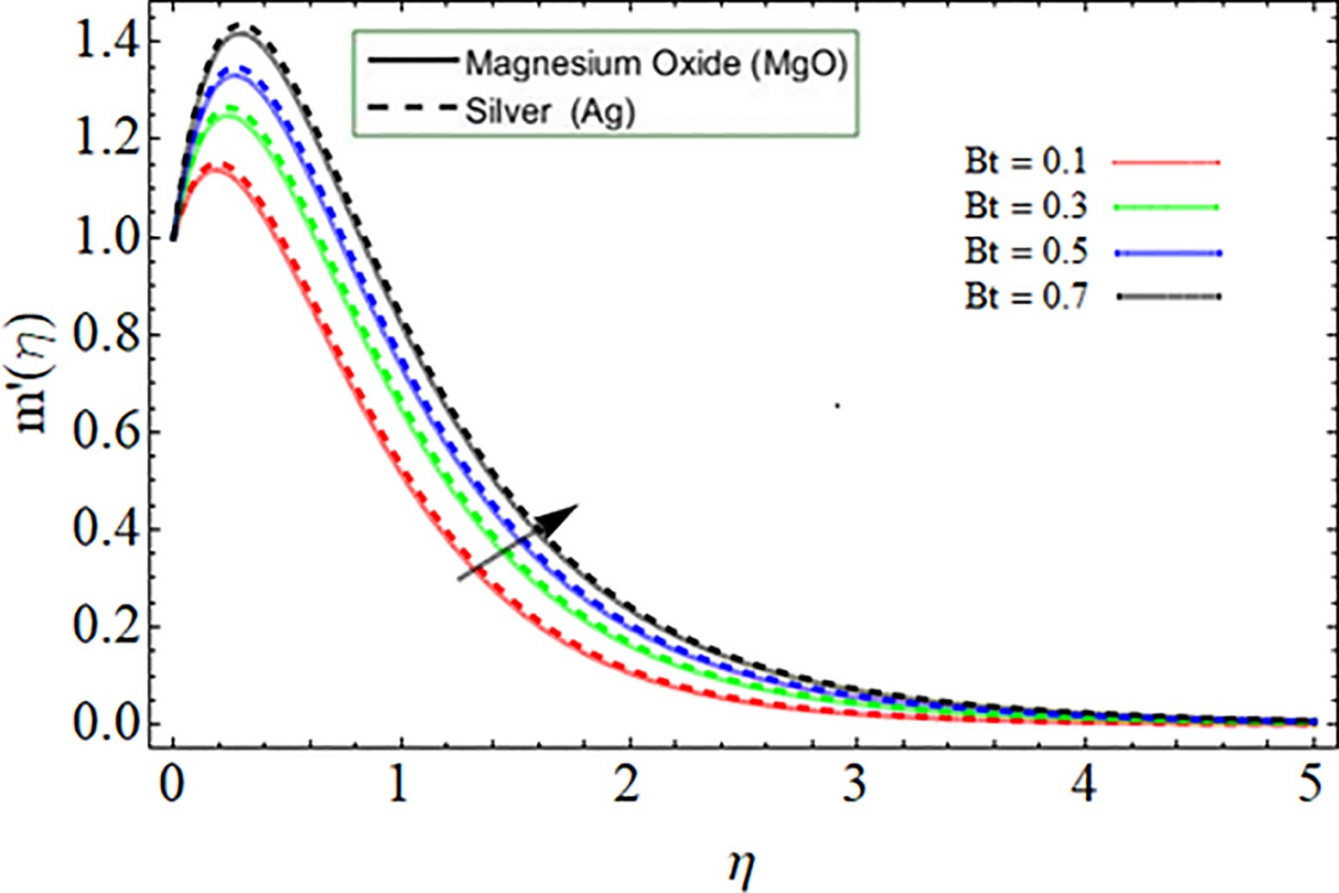


The numerical outcomes of the system of DE (Differential Equations) are calculated via using PCM, and the bvp4c technique has been used to compare and validate the results. The results have been revealed through Figs (2–11). Fig 1 displays the mechanism of fluid flow over a curvy surface with up and down fluctuation under the consequences of magnetic field. In current paper, the intention of the analysis is to boost the thermal energy transmitting rates for a range of industrial implementations. When compared to a flat surface, energy transition is increased up to 15% due to the wavy swirling surface.

### 4.2 Discussion of results

Figs (2–5) elaborate the upshot of volume friction parameters (*ϕ*_1_ or *ϕ*_*Ag*_ and *ϕ*_2_ or *ϕ*_*MgO*_) on axial velocity *f*(*η*) and energy profile *θ*(*η*) respectively. It can be perceived that the velocity and temperature transmission rate enhance with the rising values of *ϕ*_1_&*ϕ*_2_. Physically, the increasing number of nanoparticles (*Ag-MgO*) in base fluid reduces the specific heat capacity of water and improve the thermal diffusivity, because the specific heat capacity of silver and magnesium-oxide is less than water. Eventually, fluid losses its viscosity, which cause rises in temperature and velocity of fluid.

The unsteadiness parameter *S* upshot on axial *f*(*η*) and azimuthal velocity *h*(*η*) profiles is demonstrated through Figs 6 & 7, respectively. It has been concluded that the axial velocity enhances with the positive increment of unsteadiness parameter *S*, while azimuthal velocity reduces. Because the kinetic viscosity of fluid deceases with its effect, that’s why such scenario has been observed in Fig 6. The effects of disk rotation *ω* parameter on radial velocity *g*(*η*) is shown via Fig 8.

It’s reasonable to assume that improvement rate of disk rotation significantly encourages the kinematic energy of the fluid ions, that leads to an increase in velocity *g*(*η*), which also generates heat. The disc surface eventually heats up and improves the fluid temperature *θ*(*η*). Fig 9 revealed that the effect of parameter *γ* reduces the temperature profile. Because the fluid particle releases thermal energy during upward/downward movement, which causes the declination of fluid temperature. The rotating disk’s upward and downward velocity is regulated by the parameter *γ*.

**Fig 12:**
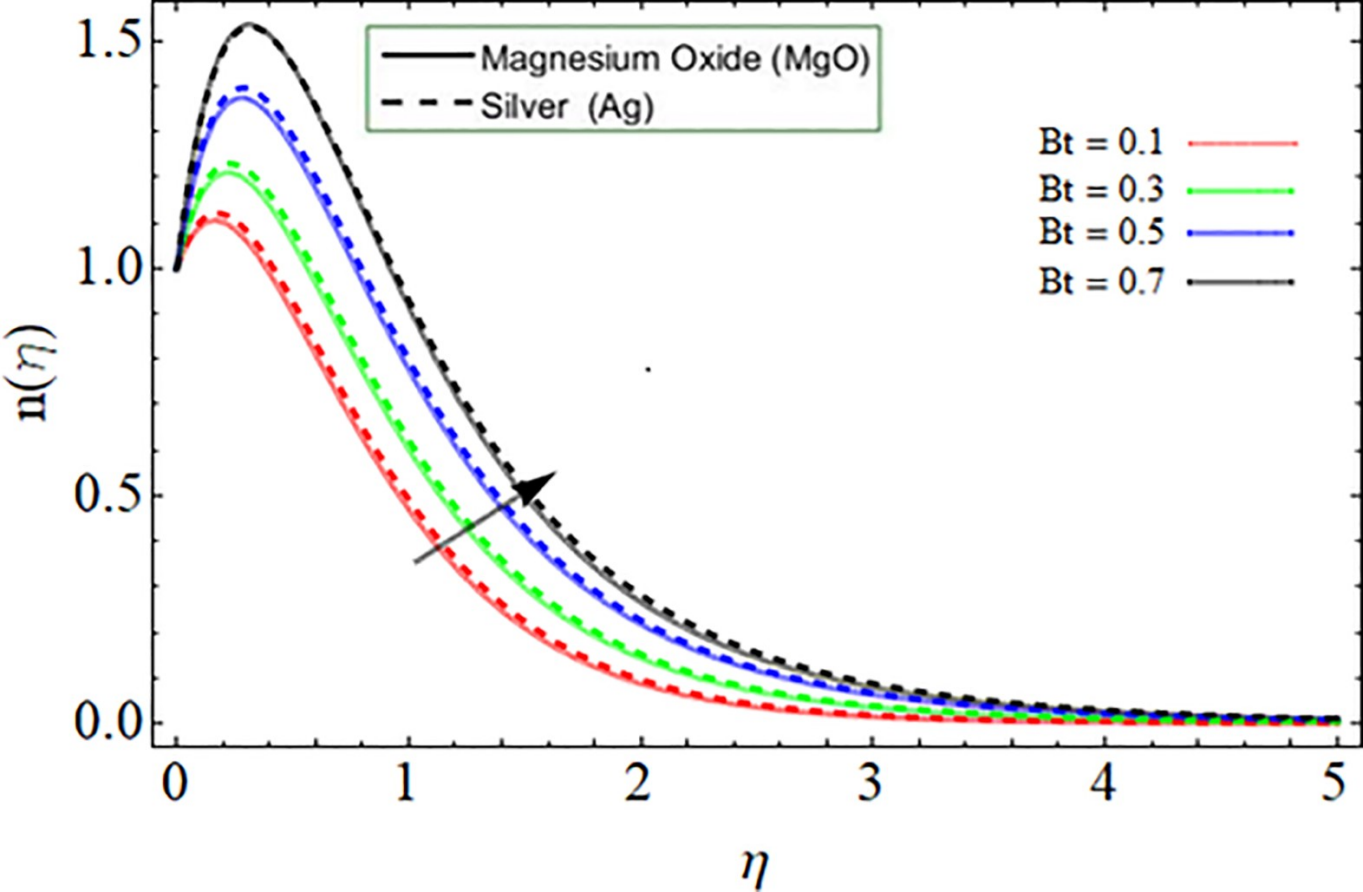


Fig 10 explored the consequences of Prandtl number *Pr* on energy profile. It can be noticed that the temperature declines by the action of Prandtl number. Physically, the action of Prandtl number directly affect the dynamic viscosity and specific heat capacity of fluid, as result the fluid thermal capability declines. Figs 11 & 12 demonstrate the influence of Batchlor number *Bt* on magnetic strength profile along axial *m*’(*η*) and radial direction *n*(*η*), respectively. The magnetic strength profile show declination versus the influence of Batchlor number, because the increment in *Bt* negatively affect the molecular diffusion rate of fluid.

Table 1 elaborate the thermophysical characteristics of silver, base fluid, and magnesium-oxide. Table 2 revealed the comparison between PCM and bvp4c method numerically for all velocities. It can be presumed that the parametric continuation method has strong convergence versus other numerical method. Tables 3 and 4 display the influence of nanoparticles quantity *ϕ* on velocity radial and azimuthal direction, skin fraction and Nusselt number.

**Table 2:** Comparative analysis between PCM and bvp4c method.

	PCM			bvp4c		
*η*	*f*(*η*)	*g*(*η*)	*θ*(*η*)	*f*(*η*)	*g*(*η*)	*θ*(*η*)
0.0	0.0000	0.0000	1.0000	0.0000	0.0000	1.0000
0.5	0.0002	0.0020	0.2711	0.0002	0.0020	0.2721
1.0	0.0060	0.0157	0.0572	0.00061	0.01488	0.0572
1.5	-0.0391	-0.0771	0.0093	-0.0393	-0.0775	0.0097
2.0	-0.1359	-0.1913	0.0019	-0.1360	-0.1914	0.0008

**Table 3:** The comparative analysis of silver and magnesium-oxide nanoparticles on velocity profile.

	Silver (Ag)		Magnesium oxide *MgO*	
*η*	*f*′(0)	*g*′(0)	*f*′(0)	*g*′(0)
0.00	1.3725	1.5912	1.4323	1.6562
0.05	1.5134	1.6724	1.8735	1.7821
0.01	1.7642	1.8054	2.1012	1.8922
0.15	2.0400	2.1791	2.2700	1.2901
0.20	2.2531	2.3753	2.4531	2.5102

**Table 4:** Quantitively analysis for Nusselt number and Skin fraction.

	Silver (Ag)		Magnesium oxide *MgO*	
*η*	*h*′(0)	*θ*′(0)	*h*′(0)	*θ*′(0)
0.00	1.2724	1.6954	1.5513	1.6582
0.05	1.2921	1.4362	1.3835	1.7734
0.10	1.5139	1.2482	0.1012	1.7622
0.15	0.3612	1.7014	1.3910	2.3684

## 5. Conclusion

In the present study, we numerically scrutinized an unsteady 3D numerical model for *Ag-MgO* hybrid nanoliquid flow with mass and energy transition caused by the up and downward fluctuation of a wavy gyrating disc. Magnesium oxide and silver nanoparticles have been heavily reported to have broad-spectrum antibacterial operations among metal oxides and metals. However, in current paper, the study’s goal is to increase the rate of thermal energy propagation for a variety of industrial applications. For the purpose, the problem has been formulated as a system of partial differential equations (Navier Stokes and Maxwell). During the study, the following observation have been made:

- Silver nanoparticles are without a doubt the most used inorganic nanoparticles, with numerous innovations in biomaterial’s detection and antimicrobial operations.
- In comparison to a smooth substrate the wavy spinning surface raises the heat transfer rate by 15%.
- Magnesium oxide (MgO) is made up of *Mg*^2+^ and *O*^2-^ ions that are held together by a strong ionic bond. Which can be synthesized at temperatures ranging from 700 ^0^C to 1500 ^0^C degrees Celsius and is primarily used in refractory and electrical applications.
- The up and downward fluctuation of the curly spinning disc affects fluid temperature and velocity in a constructive way.
- Water’s thermophysical properties are improved by the close bonds between water atoms (*H*^+^+*OH*^-^) and silver ions Ag^+^.
- Hybrid nanoliquids are more effective at overcoming low energy transfer. For example, it improves carrier fluid thermal efficiency, which is essential in power generation, microfabrication, hyperthermia, metallurgical fields and air conditioning.
- The parametric continuation method is a strong numerical technique for highly nonlinear system of PDE than others numerical algorithm.
